# A Bayesian computational model to investigate expert anticipation of a seemingly unpredictable ball bounce

**DOI:** 10.1007/s00426-022-01687-7

**Published:** 2022-05-24

**Authors:** David J. Harris, Jamie S. North, Oliver R. Runswick

**Affiliations:** 1 School of Sport and Health Sciences, College of Life and Environmental Sciences, University of Exeter, St Luke’s Campus, Exeter, EX1 2LU UK; 2 Research Centre for Applied Performance Sciences, Faculty of Sport, Allied Health, and Performance Science, St Mary’s University, Twickenham, UK; 3 Department of Psychology, Institute of Psychiatry, Psychology and Neuroscience, King’s College London, London, UK

## Abstract

During dynamic and time-constrained sporting tasks performers rely on both online perceptual information and prior contextual knowledge to make effective anticipatory judgments. It has been suggested that performers may integrate these sources of information in an approximately Bayesian fashion, by weighting available information sources according to their expected precision. In the present work, we extended Bayesian brain approaches to anticipation by using formal computational models to estimate how performers weighted different information sources when anticipating the bounce direction of a rugby ball. Both recreational (novice) and professional (expert) rugby players (*n* = 58) were asked to predict the bounce height of an oncoming rugby ball in a temporal occlusion paradigm. A computational model, based on a partially observable Markov decision process, was fitted to observed responses to estimate participants’ weighting of online sensory cues and prior beliefs about ball bounce height. The results showed that experts were more sensitive to online sensory information, but that neither experts nor novices relied heavily on prior beliefs about ball trajectories in this task. Experts, but not novices, were observed to down-weight priors in their anticipatory decisions as later and more precise visual cues emerged, as predicted by Bayesian and active inference accounts of perception.

## Introduction

In sport and other time-constrained dynamic tasks, a performance advantage can be gained from predicting future outcomes and executing anticipatory actions, rather than merely reacting to unfolding events (Loffing & Cañal-Bruland, 2017). Indeed, sensory processing latencies mean that during the most time-constrained tasks, predictions are necessary for successful performance (Loffing & Cañal-Bruland, 2017; Morris-Binelli & Müller, 2017). Researchers have consistently shown that, across domains, the most skilled performers are particularly adept at making predictions (see Araújo & Kirlik, 2008; Gredin et al., 2018; Morris-Binelli & Müller, 2017; Savelsbergh et al., 2005). However, it remains unclear exactly *how* experts are able to optimally integrate and weight multiple, ever-changing, and often incomplete information sources to make effective anticipatory judgements (Cañal-Bruland & Mann, 2015; Gredin et al., 2018; Williams & Jackson, 2019). In this study, we explore how performers weight the importance of different information sources when anticipating the bounce of a ball using a Bayesian computational model based on active inference theories of perception and action (Parr & Friston, 2019; Parr et al., 2021).

## Anticipation

Skilled anticipation requires integrating multiple sources of information to make the most accurate predictions. Researchers have previously focused on two broad types of information: *online sensory cues* and *contextual priors*:

i) Online sensory cues are the sources of perceptual information that emerge during action. These cues are often visual information, such as the posture of an opponent (Savelsbergh et al., 2002), relative motion between players in team sports (North et al., 2009), or the flight path of a ball that needs to be intercepted (Croft et al., 2010). However, other sensory evidence, such as auditory cues from racquet-ball contact (Cañal-Bruland et al., 2018), can also be informative. Crucially, multiple sources of sensory information will be used to support predictions as they emerge.
ii) Contextual priors refer to a tiered hierarchy of information that is available before sensory cues emerge and which facilitate an advance understanding of the situation. For instance, a returner in tennis may know that the server directs their first serve ‘out wide’ on the majority of occasions (i.e., their action preferences; Mann et al., 2014), and can prepare for and anticipate this response. Other contextual priors include the current game score (Farrow & Reid, 2012) or opponents’ positioning on the court or field (Loffing & Hagemann, 2014). But prior knowledge extends beyond just current contextual information to wider beliefs about regularities in the environment, such as the physical properties of objects and their likely behaviors.^1^ In essence, prior beliefs include any previous knowledge that provides an indication of the marginal probability of an outcome, which then facilitates responses to more likely events.

The reliability of online sensory cues and priors is dependent on the specific task and source of information. In rugby, for example, sensory cues from an oncoming opponent (e.g., motion of head or hips) can be used to either enhance action anticipation or can be deceptive (Warren-West & Jackson, 2020). Particular game scenarios can also provide very reliable contextual information. For instance, when a player is in possession of the ball, they have good kicking ability, their team is winning, and time has expired on the clock, there is a high probability that they will kick the ball out of play to win the game. By contrast, if the player kicked the ball towards you and it bounced, there is little contextual information about which way it will bounce (Runswick et al., 2020b). You may, however, still hold some more general prior beliefs about how high a rugby ball typically bounces that help to guide your anticipation, in addition to relying on online sensory cues from ball flight.

Theories of expert performance have proposed that extended and focused practice enables elite athletes to develop extensive domain-specific knowledge structures (Ericsson & Kintsch, 1995; Yarrow et al., 2009). This domain-specific knowledge provides an understanding of relevant context but also guides sensitivity to critical cues, the planning of actions, continual evaluation of the present situation, and prediction of future outcomes. Consequently, expert performers not only have access to more information to guide anticipation but can create enhanced representations of the current environment by facilitating the integration of environmental information with existing representations (e.g., Ericsson, 2000). However, a greater quantity of information is not the only reason for superior anticipation; experts are also adept at applying this information. For instance, experts are less likely to be misled by incongruent information, as they are able to identify and down-weight deceptive cues (Jackson et al., 2020). It is this integrating and weighting of different information sources, not just in elite athletes but all performers, that is still to be fully understood (Gredin et al., 2020).

## Internal predictive models and active inference

**Fig. 1:**
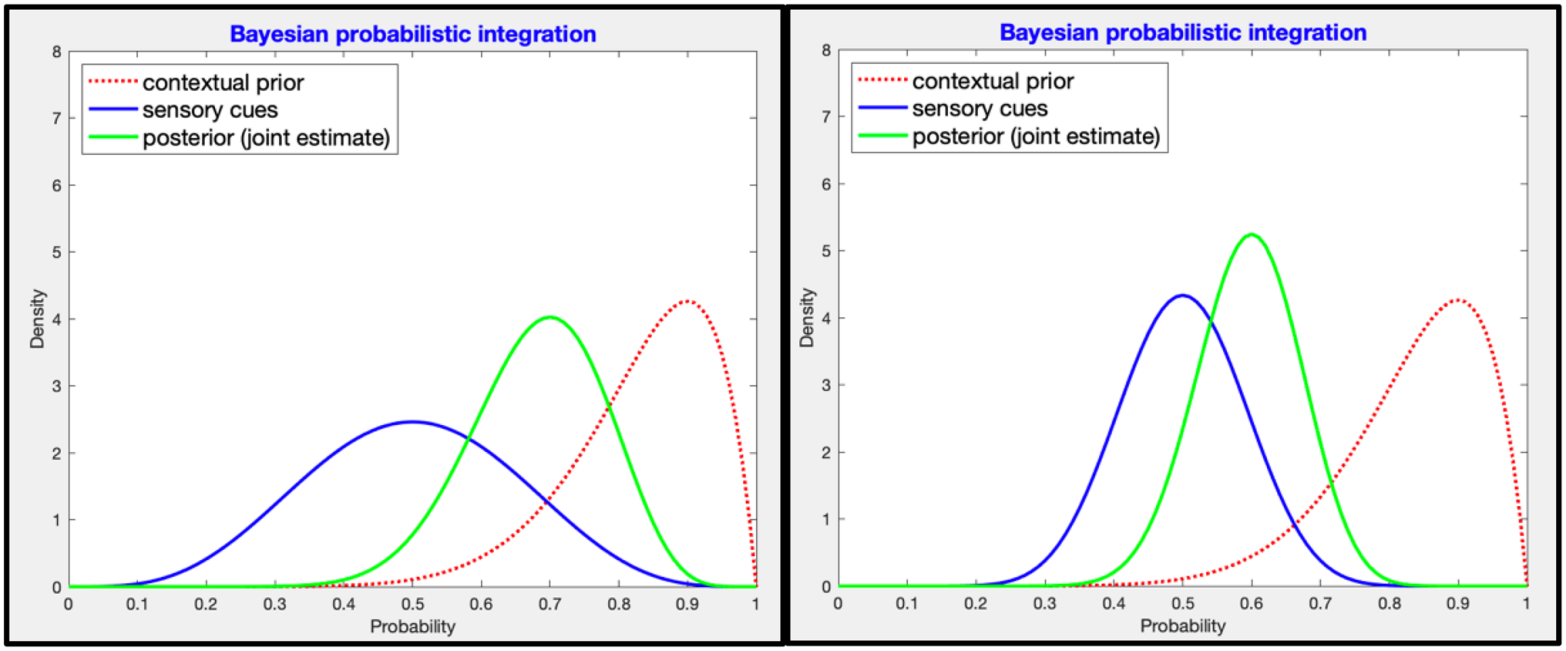
Illustration of Bayesian probabilistic integration. Beta density plots show the principle of integrating information sources according to their precision. The posterior belief is a joint estimate of the prior belief and the sensory information (likelihood distribution). On the left, the sensory cues are centered on *p* = 0.5, but are relatively weak/imprecise (wider distribution). On the right, the sensory cues distribution is still centered on *p* = 0.5 but is more certain, hence the posterior is pulled closer towards the sensory cues distribution

Researchers have recently proposed that ‘Bayesian brain’ accounts of how individuals integrate multiple information sources during perception and decision-making can be applied to understand anticipation in sport (Gredin et al., 2018, 2020; Harris et al., 2021; Helm et al., 2020). The Bayesian brain is a formal account of how rational agents should reason and make perceptual inferences in situations of uncertainty (Clark, 2013; Knill & Pouget, 2004). Uncertainty is inherent in all perceptual judgements, arising from noisy sensory feedback, imperfect knowledge of the world, and a changing environment. In the Bayesian brain, information is represented as probability distributions and information sources are combined according to their (un)certainty, such that information that is believed to be more precise (e.g., those based on extensive experience) will be afforded greater weight in computations (see Fig. 1). Consistent with this proposal, human observers have been found to integrate information in an approximately Bayes-optimal way (i.e., will minimize error in a probabilistic manner) in many tasks (Behrens et al., 2007; Knill & Pouget, 2004; Körding & Wolpert, 2006; Yu, 2007). For instance, when attempting to estimate the position of a mouse cursor relative to their hand, participants were observed to increasingly rely on knowledge developed over previous trials (their prior) when the current visual information was occluded to a greater degree (i.e., more uncertain) (Körding & Wolpert, 2004). This re-weighting of information during the perceptual judgment was consistent with the strategy predicted by optimal Bayesian calculations.

Bayes-optimal decision-making is based on developing a generative (predictive) model of the world, which can be used to make predictions about events or sensory stimulation (X is likely to lead to Y) or can be *inverted* to infer the hidden cause of some observation (Y was most likely caused by X). For human perception, the brain relies on a model of the causal relationships among (hidden) states of the world that produce sensory input in order to make inferences about the causes of its sensations (Friston, 2005, 2010). When predictions are inaccurate, the generative model is revised to improve future predictions. Predictive coding (Rao & Ballard, 1999) describes how this error signaling is achieved at a neuronal level: descending cortical projections encode predictions and ascending connections signal deviations from those predictions, which serve to revise subsequent predictions at higher levels of the cortical hierarchy (Shipp et al., 2013). Consequently, Bayesian agents always seek to minimize the error of their predictions (also known as surprisal; Baldi & Itti, 2010), thereby maximizing the predictive value of their generative model of the world.

Active inference extends predictive coding models of perception to the use of action to minimize future prediction errors (Friston, 2005; Parr & Friston, 2018, 2019). In addition to constantly revising their generative model, a Bayesian agent can minimize prediction errors through movements (e.g., see the use of vision to minimize surprisal; Arthur & Harris, 2021; Parr et al., 2021) or can actively change the world into the predicted state (Adams et al., 2013; Sarpeshkar et al., 2017). Harris et al. (2021) have recently suggested that active inference can enhance our understanding of skilled anticipation by providing a principled account of how actions are used to optimize predictions, as well as accounting for decision-making via Bayesian inference. However, such theorizing about the use of action to optimize predictions is largely missing from current accounts of anticipation, as identified in Runswick et al. (2020a, 2020b) framework.

An additional benefit of Bayesian brain and active inference frameworks is that they are rooted in computational models of perceptual processes (Adams et al., 2015; Parr et al., 2021; Smith et al., 2020). These models formalize active inference, and the process of Bayesian belief updating, making mechanistic explanations potentially clear and testable. For current purposes, applying computational models to empirical data also enables us to estimate the relative weightings that individuals apply to different informational sources. This affords an opportunity to examine the effect of factors like expertise and visual occlusion on the relative weightings of priors and sensory cues. Therefore, in the present study, we adopted a computational active inference model of perception to investigate the anticipatory behavior of novice and expert rugby union players when predicting the bounce of a rugby ball.

## The present work

The future trajectory of a bouncing rugby ball is notoriously hard to predict. Due to its oval shape, the reaction force can act ahead of or behind the center of the ball and hence, when projected with topspin, the ball can either roll before bouncing or suddenly bounce to a much greater height. However, the highly variable bounce profiles do correspond to describable physical laws (see Cross, 2010) and therefore experts (with extensive domain knowledge) may have prior beliefs about likely bounce profiles that enhance their predictions about bounce height. Consistent with this proposal (Runswick et al., 2020a) demonstrated that task experts (professional rugby players) were better able to predict the bounce of an oncoming rugby ball than novice participants. Using a temporal occlusion method, Runswick et al. (2020a, 2020b) reported that both novice and expert groups anticipated ball bounce more accurately when more sensory cues were available (the ball flight and postural cues from the kicker), and that expert differences were most pronounced for early occlusion (postural cues only). Active inference accounts of anticipation (Harris et al., 2021) explain the underlying mechanism of these effects based on the developed generative model of the expert performer and the relative contribution of less certain sensory cues (i.e., during visual occlusion). However, active inference models are yet to be explicitly applied to empirical data during anticipation in sport. Therefore, we aimed to extend the active inference approach to anticipation (Harris et al., 2021) by scrutinizing the relative contributions of online sensory information and prior beliefs during expert anticipation. To this end, we performed a reanalysis of the data reported in Runswick et al. (2020a) by fitting a Bayesian computational model to the anticipatory behavior of novice and expert players to determine the role of priors (in the form of prior knowledge about the behaviors of a bouncing oval ball) and sensitivity to sensory information (in the form of the opponent’s kinematics during the kick and the kinematics of the ball during flight) in their anticipation performance.

### Hypotheses

H_1_—Experts will be able to make better use of both ball bounce priors and sensory cues:

While no explicit contextual information (e.g., game score) was provided in this task, experts should have a more developed generative model of the ball bounce task based on their extensive experience. Consequently, they will have stronger prior expectations about the most likely bounce trajectories, as well as an established mapping between sensory cues and likely outcome states that will enable them to be more attuned to sensory information.

H_2_—Experts will rely more on priors when sensory information is less reliable:

Both groups should, if integrating information in a Bayesian fashion, rely more strongly on prior predictions when sensory information is less certain (i.e., when it is occluded earlier). However, as novices are likely to be insensitive to the sensory information in this task anyway, it is expected that this effect will only be present in experts (Gredin et al., 2018).

## Methods

### Preregistration

Following data collection, but prior to data analysis, we pre-registered the primary hypotheses and planned analyses on the Open Science Framework, which can be viewed here: https://osf.io/x64bh/. Any deviations from the analysis plan are specified as exploratory.

### Design

This study adopted a mixed design, with independent groups for skill level (expert and novice), and repeated measures for temporal occlusion conditions (postural cues only; ball flight only; postural cues and ball flight) and kick type (chip or grubber kick).

### Participants

Fifty-eight participants took part in the experiment; the expert group consisted of 38 professional rugby union players (*M*_age_ = 25.9 ± 3.4 years; *M*_experience_ = 11.9 ± 6.8 years) while the novice group consisted of 20 less-skilled players (*M*_age_ = 22.4 ± 3.6 years; *M*_experience_ = 1.9 ± 2.2 years). At the time of recruitment, all participants in the expert group were competing in the English Championship (the second tier of professional rugby) and reported a mean weekly playing time of 13.5 ± 8.4 h at that level. Of the expert participants, 17 had prior playing experience at Premiership or International level rugby. The less-skilled participants had no history of competitive rugby beyond recreational participation and compulsory school classes. The study received ethical approval from the lead University ethics committee. All participants provided fully informed written consent prior to taking part.

As this study was a reanalysis of an existing dataset (described in Runswick et al. (2020a) and no a priori power calculation was possible, we generated a series of power curves for a range of effect sizes to determine the effects that we were powered to detect. Power curves were generated using the R package ‘simR’ (Green & MacLeod, 2016), and were based on the known variance of the existing data. For the primary effect of group in a mixed-effects model, with participant as a random factor, power curves indicated that the current sample provided > 95% power for a large-to-medium effect of std. beta = 0.5, ~ 85% power for a medium effect of std beta = 0.4, and ~ 65% power for a small-to-medium effect of std beta = 0.3. The power curves, and R code for generating them, are available from the online supplementary files (https://osf.io/x64bh/).

### Task and materials

The experimental task consisted of a video anticipation test with temporal occlusion in which participants were asked to predict the future location of a bouncing rugby ball. Video stimuli featured a right-footed rugby union player (University First XV) performing two kick types: ‘grubber’ and ‘chip kicks’. Grubber kicks refer to when the ball is struck to roll along the ground, mostly rotating end over end. Chip kicks are struck so as to travel in a high arc and bounce only once. The kicks were performed on a grass rugby field with a size five rugby ball (Gilbert Photon), from a distance of 15 m from the camera. Videos were recorded with a Panasonic HC-V210 HD camcorder at 50 Hz (Panasonic UK Ltd., Berkshire, UK) set at eye-level at a height (1.7 m). Clips of 23 grubber kicks and 14 chip kicks were edited to occlude the video at three different time points: (i) occlusion immediately prior to ball-foot contact (postural cues only—PC); (ii) occlusion at the last frame of ball-to-ground contact (postural cues and ball flight—PC&BF); and (iii) occlusion before the point of foot-to-ball contact and after the last frame of ball-to-ground contact (ball flight only—BF). These occlusion conditions represented different levels of precision in the available sensory information. Combined postural cues and ball flight provided the most precise and informative cues. Postural cues provided only the earliest information and therefore possibly the least precise information, while ball flight provided slightly later and therefore possibly more precise information. No additional contextual information (e.g., game situation) was provided. Clips were repeated across each of the three occlusion points to account for any variation in trial difficulty. The resultant 111 video clips (mean length of 2.4 ± 1 s) were put together in a randomized order into one 20-min video. The video was then checked to ensure that clips repeated across conditions were not displayed sequentially. To maintain the representativeness of the footage (i.e., to allow experts to make use of any ball bounce priors) the distribution of kick outcomes was not controlled. Instead, all kicks that bounced within a 4 m × 4 m target area were included, which resulted in 48 low bouncing and 21 high bouncing grubber kicks, and a further 21 for each of high bouncing chips kicks and low bouncing chip kicks.

**Fig. 2:**
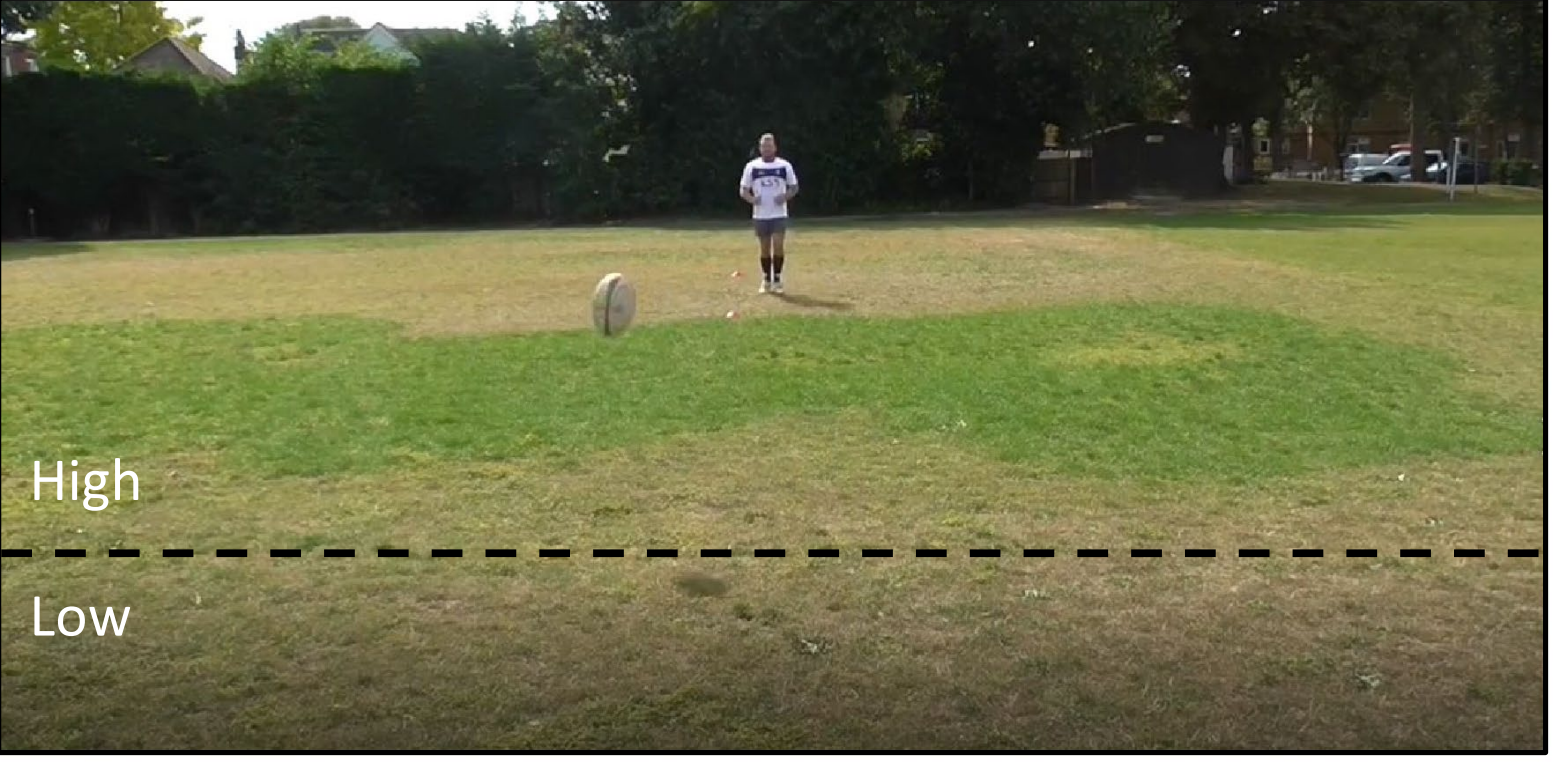
Screenshot from the video anticipation task. This image is taken from one of the stimulus videos in the experiment. Kicks were executed in the direction of the camera from 15 m away and then projected on a screen measuring 5 × 3.5 m. The image shows the ball in flight after being kicked, and the demarcation between a ‘high’ (meaning participants would receive the ball at chest height or above) and a ‘low’ (meaning participants would need to lower their chest to receive the ball) bounce, that participants were asked to predict

The video anticipation test was displayed via a projection (Sanyo PDG-DET100L Projector; Sanyo Electric Co Ltd., Osaka, Japan) onto a white wall to create a large 5 m × 3.5 m image. Participants were instructed to predict the direction that they believed the ball would bounce. The original study by Runswick et al. (2020a) recorded predicted bounce direction in horizontal (left, middle, or right) and vertical (high or low) directions. However, as no performance effects were reported for the horizontal direction, we only modeled the responses in the vertical direction. Vertical bounce location was defined in relation to whether the ball-bounced into the top two thirds of the screen (approximately equivalent to receiving the ball at chest height or above), or the bottom third (approximately equivalent to needing to reach down to intercept the ball; see Fig. 2). Additional details of the task can be obtained from Runswick et al. (2020a).

### Procedure

All participants had the purpose of the experiment explained to them and provided written informed consent prior to taking part in the experiment. Next, participants viewed a familiarization video which consisted of the same process as one experimental trial for each trial possibility (i.e., six trials showing chip and grubber kicks for all three occlusion possibilities). Participants then completed the video anticipation test by marking their prediction about bounce location using pencil and paper and a response grid. The test was completed either alone or in small groups. During small group testing, participants were seated apart and square on to the screen.

### Computational model

**Fig. 3:**
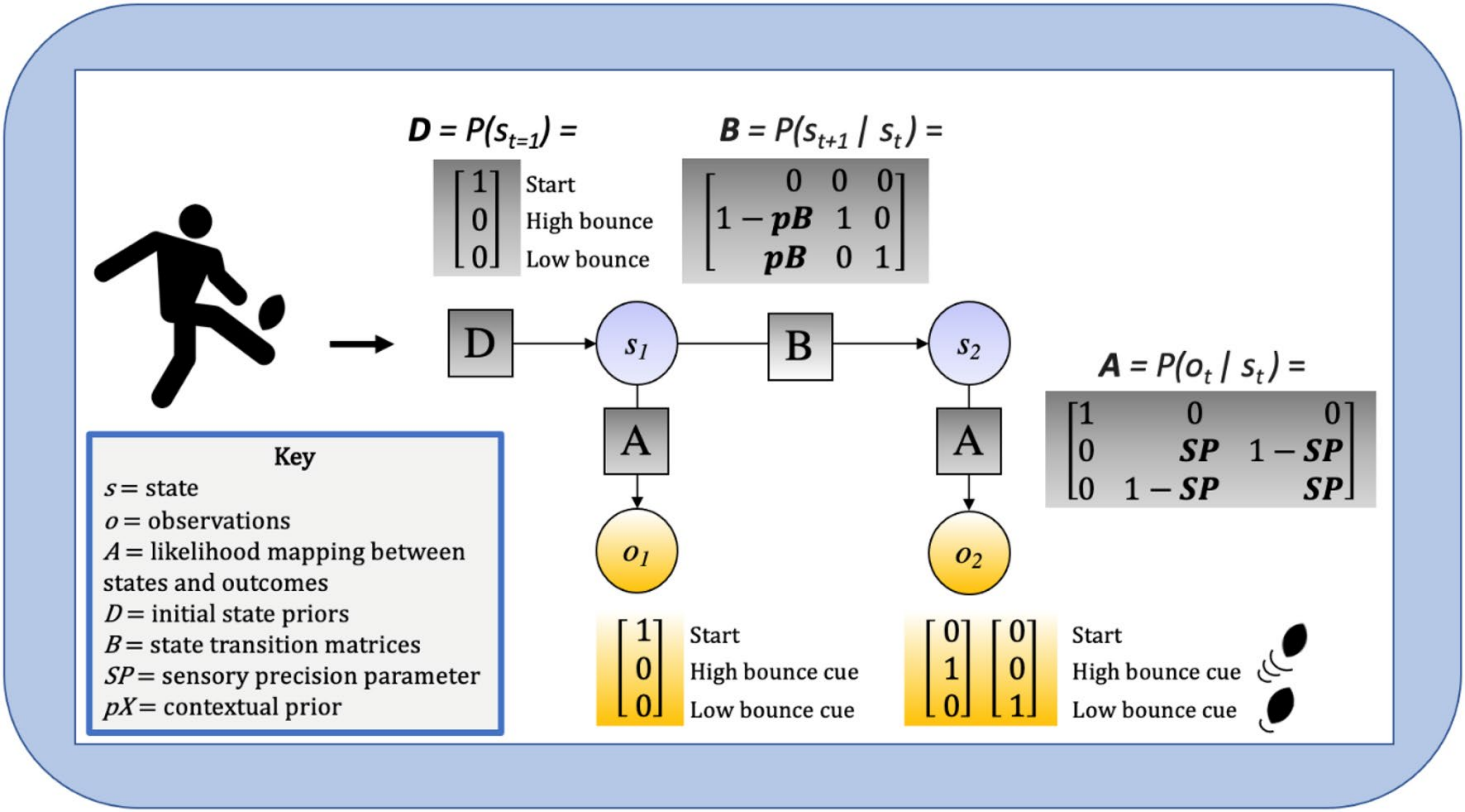
Bayesian network representation of perceptual inference during ball bounce anticipation. The POMDP generative model is depicted graphically; arrows represent dependencies between variables, circles (‘nodes’) correspond to variables (states and observations) and squares represent factors mediating the conditional relationships. At each time point (*t*), observations (*o*) depend on hidden states (*s*), where this relationship is specified by the A matrix which maps the likelihood of states given observations. Those states, in turn, depend on previous states (as specified by the B matrix, or the initial states specified by the D vector). Subscripts for states and observations indicate time points within a trial (*τ*)

To model anticipatory behavior in the ball bounce anticipation task, we adopted a Bayesian model of perception derived from a partially observable Markov decision process (POMDP) (Da Costa et al., 2020; Friston et al., 2017; Smith et al., 2020, 2021). To avoid the computational model ‘black box’ (Stafford, 2009) we provide a detailed description of all elements of the model (and all code: https://osf.io/x64bh/). Put simply, the model performs Bayesian inference through combining prior beliefs with observations according to their respective precisions (e.g., Fig. 1) to produce a posterior belief about the true state of ball bounciness. As participants’ beliefs following each trial were known from their responses, we performed gradient descent over two free parameters in the model to optimize estimates for prior beliefs (pB) and sensory precision (SP) that maximized the predictive power of the model, given the known responses. The estimated pB and SP parameters were then model outputs which we compared between groups and conditions. Figure 3 provides a graphical description of the model and associated vectors and matrices.

Each trial in the POMDP model was formalized as consisting of two timesteps (*t* = 1 and *t* = 2). At *t* = 1, the participant always began in the “start” state and made the associated “start” observation. At *t* = 2, the participant observed visual cues from the video stimulus and inferred whether they had transitioned into the high bounce state or the low bounce state. That is, they inferred a posterior distribution over *P*(*s*_*t* = 2_) that assigned a probability to the high versus low state, based on a Bayesian integration of prior beliefs about transitioning to the respective states *P*(*s*_*t* = 2_|*s*_*t* = 1_) and beliefs about the likelihood of observing a high cue or low cue given an underlying high or low state, *P*(*o*_*t*_|*s*_*t*_) (i.e., the likelihood mapping). Observations in the model (denoted *o*) were categorical and consisted of a “start” observation, a high bounce cue observation, and a low bounce cue observation. In the task, the ‘high/low bounce cue observation’ consisted of all the early sensory indicators that might predict a high or low ball bounce, such as the kinematics of the kicker and the flight of the ball. These observations provided only partial information about bounce outcome but will be referred to as the ‘high/low bounce cue’ from hereon. Hidden states (denoted *s*) in the model (also categorical), were a “start” state, a “high bounce” state, and a “low bounce” state. Hidden states are inferred from observed instances, mediated by the *A* matrix, which is illustrated by the dependency arrow in the figure.

The vector *D* encoded prior beliefs over initial states, *P*(*s*_*t* = 1_), which specified that the participant always started the trial in the “start” state and was certain in their belief. The matrix *B* encoded the probability of each state transitioning into any other between timepoints:

$$B = P\left( {s_{{t = 2}} |s_{{t = 1}} } \right) = \left[ {\begin{array}{*{20}l} 0 & 0 & 0 \\ {1 - {\text{pB}}} & 1 & 0 \\ {{\text{pB}}} & 0 & 1 \\ \end{array} } \right]$$

Columns (left to right) denote the start state, the high state, and the low state at time *t* = 1, and rows (top to bottom) denote the start state, the high state, and the low state at time *t* = 2. The parameter pB, therefore, represents the probability of transitioning from the start state to the high state—with values above 0.5 showing prior beliefs that transitions to the low bounce state are more likely and those below 0.5 showing that high bounce states are more likely. Determining this parameter for each participant therefore indicates the direction and strength of their belief about moving to each state—effectively their prior beliefs about bounciness. The second and third columns encode that once entering a high or low state the state does not change within that trial.

The *A* matrix mapped the probability of possible observations given hidden states, through which the participant could infer the high or low state from the observed cues. This equates to the inversion of the agent’s generative model where they infer likely causes from observed stimuli. Formally:

$$A = P\left( {o_{t} |s_{t} } \right) = \left[ {\begin{array}{*{20}l} 1 & 0 & 0 \\ 0 & {{\text{SP}}} & {1 - {\text{SP}}} \\ 0 & {1 - {\text{SP}}} & {{\text{SP}}} \\ \end{array} } \right].$$

Columns (left to right) denote the “start” state, the high bounce state, and the low bounce state, and rows (top to bottom) denote the “start” observation, the high bounce cue observation, and the low bounce cue observation. The probability of observing a high or low cue, given a high or low state was encoded by a “sensory precision” parameter (SP). An SP value of 0.5 indicates minimal precision (no relationship between the cue and hidden state), while a value approaching 1 suggests high precision (the probability of observing a high bounce cue is high in a high bounce state and low when in a low bounce state (and vice versa for values approaching 0 for low bounce). In behavioral terms, SP values approaching 1 would indicate that a participant consistently responded ‘high’ when a high cue was present.

Bayesian belief updating in the model was based on the following equations for the two timepoints (*t* = 1 and *t* = 2, respectively):

$${\overline{{\varvec{s}}} }_{{\varvec{t}}=1}={\varvec{\sigma}}\left(\begin{array}{l} \\ \frac{1}{2}\left(\mathrm{ln}D+\mathrm{ln}B \bullet {s}_{t+1})+\mathrm{ln }A\bullet {o}_{t}\right.\\ \end{array}\right),$$

$${\overline{{\varvec{s}}} }_{{\varvec{t}}=2}={\varvec{\sigma}}\left(\mathrm{ln}B {s}_{t-1}+\mathrm{ln }A\bullet {o}_{t}\right).$$

Here, ${\varvec{\sigma}}$ indicates a SoftMax function which converts the belief to a proper probability distribution and ln refers to the natural logarithm. In essence, beliefs at *t* = 2 are a function of integrating SP and pB based on their precision, according to Bayes’ rule. Our model assumes that the probability of selecting a high or low bounce response was directly related to the posterior distribution over states at time *t* = 2 in each trial, such that choices to select high increased as the posterior over high approached 1 (i.e., *P*(high) = *P*(*s*_*t* = 2_ = high)). The observed responses of participants were modeled using the Bayesian model of perception described here.

The target parameters (SP and pB) within the model were then estimated using a Bayesian optimization algorithm (called Variational Bayes) which identifies the parameter values that maximize the likelihood of the participants responses (e.g., see Schwartenbeck & Friston, 2016). Parameter estimation first required setting prior means and variances for each parameter. We set a high precision value of 1/2 for each parameter to deter overfitting and work from prior means SP = 0.5 and pB = 0.5. We first ran a POMDP model to estimate overall SP and pB parameters for novice and expert groups, then ran a second model to estimate SP and pB across all kick type and occlusion conditions. Parameter recoverability analyses indicated that the model was sensitive to changes in the true parameters, such that artificially inputted true parameters were highly recoverable for both *SP* (*R*^2^ = 0.84, *p* < 0.001) and pB (*R*^2^ = 0.82, *p* < 0.001) (see supplementary files for plots). It should be noted that the SP and pB are somewhat arbitrary values on the scale of 0–1 which are indicative of relative strength and direction of beliefs for modeling purposes (Table 1).

### Data analysis

Computational modeling was performed in MATLAB R2019a (Mathsworks, MA) and statistical analysis in RStudio v1.0.143 (R Core Team, 2017). Parameter estimates were retrieved from the POMDP models and then screened for outlying values (> 3 standard deviations from the mean; Tabachnick & Fidell, 1996). Outliers were replaced with a Winsorized score by changing the outlying value to a value 1% larger (or smaller) than the next most extreme score (6 values; < 1%).

A linear mixed-effects model was used to examine the effect of group (novice vs expert) and occlusion condition (PC vs BF vs PC&BF) on the model outputs. Models were run using the lme4 package for R (Bates et al., 2014). As specified in the pre-registration, a ‘maximal’ model was initially run, with all possible random factors for participants and condition (Barr et al., 2013). A Principal Components Analysis was then used to identify which random factors explained additional variance, and factors with little explanatory power were removed from the model to avoid overfitting, as described in Bates et al. (2018). The Akaike information criterion was then used to compare models and ensure that the simplified model did indeed provide a better fit to the data. When interpreting the results of mixed-effects models, we followed the rules of thumb outlined in Acock (2014), who suggested that standardized beta effect sizes can be interpreted similarly to r (i.e., < 0.2 is weak, 0.2–0.5 is moderate, and > 0.5 is strong). A standardized beta of 0.5 indicates that a one standard deviation change in the predictor variable equates to a half standard deviation change in the outcome variable.

Bayes Factors (using JZS priors) for mixed-effects models were calculated using the BayesFactor package (Morey & Rouder, 2015) to provide more informative conclusions about null effects and derive conclusions not based on a single approach. We report BF for main effects and overall interactions, which denotes the probability of the data under the alternative hypothesis (against the intercept-only null); values greater than one (> 1) indicate the alternative to be the more likely model, while values less than one (< 1) indicate the null to be more likely. All analysis scripts are available in the supplementary materials from the Open Science Framework: https://osf.io/x64bh/.

## Results

### Effect of expertise

**Fig. 4:**
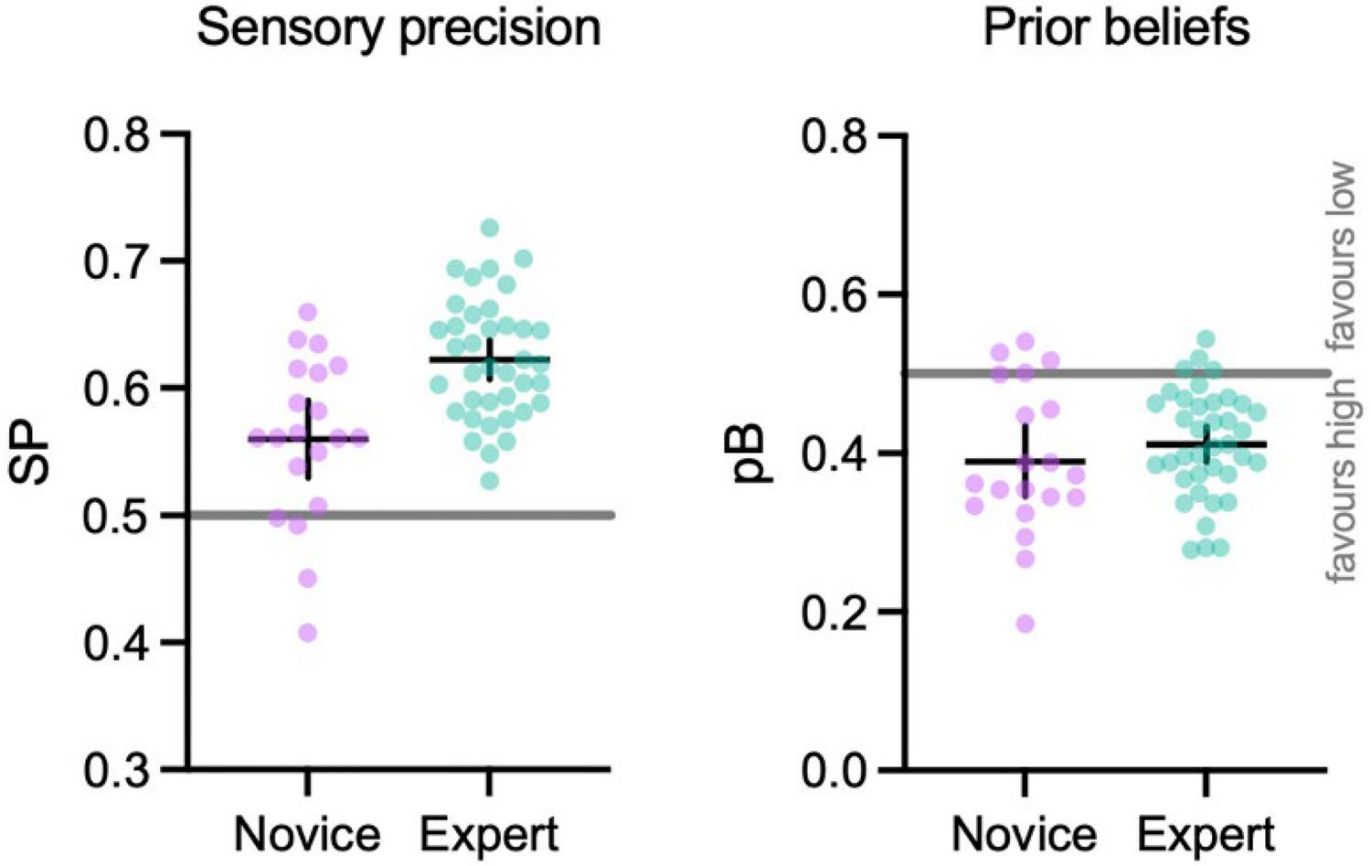
Grouped scatterplot of sensory precision and prior belief parameters (with means and 95% CIs). SP values around 0.5 indicate that there was little mapping between observations and beliefs about hidden states (i.e., insensitive to online cues) while values approaching 1 indicate increasing sensitivity. pB values above 0.5 indicate a prior belief that low bounces are more likely to occur (with values closer to 1 indicating stronger beliefs), and vice versa for values below 0.5

To test whether experts were more sensitive to sensory cues and held stronger priors during anticipation (H_1_), we analyzed the parameter estimates taken from the overall POMDP, using a simple linear model. The linear model explained a significant and large proportion of variance in sensory precision scores (*F* (1, 56) = 17.69, *p* < 0.001, *R*^*2*^ = 0.23). The model's intercept was at 0.62 (SE = 0.01, 95% CI [0.60, 0.64], *p* < 0.001). Within this model the effect of expertise was very large and significant (beta = -0.06, SE = 0.01, std. beta = 1.02, *p* < 0.001, BF = 229.07), with experts displaying higher sensory precision values (see Fig. 4). However, a second model indicated that there was no overall difference between novices and experts in the use of priors (*F* (1, 56) = 0.99, *p* = 0.32, *R*^2^ = 0.02). The model's intercept was at 0.41 (SE 0.01, 95% CI [0.39, 0.44], *p* < 0.001), with the effect of group being small and not significant (beta = − 0.02, SE 0.02, std. beta = − 0.27, *p* = 0.32, BF 0.42) (see Fig. 4). Model fit checks are available in the supplementary files.

### Effect of occlusion

To examine whether experts relied more heavily on priors, and less on sensory cues, when sensory inputs were more uncertain (i.e., during earlier occlusion) (H_2_) we examined SP and pB parameters across conditions and kick types. We fitted a linear mixed model (estimated using maximum likelihood) predicting sensory precision values from group and occlusion condition. The final model simply included ‘participant’ as a random effect (i.e., random intercepts). The total explanatory power of the model was large (*R*^2^ = 0.19) with the fixed effects alone explaining 10% of the variance. The model's intercept (corresponding to SP = 0, group = Expert, and Occlusion = PC), was at 0.55 (SE 0.01, 95% CI [0.54, 0.57], *p* < 0.001). Within this model there was a medium sized, and significant, effect of group (beta = − 0.05, SE = 0.02, std. beta = − 0.57, *p* = 0.003, BF = 2.29 × 10^4^). Relative to the reference category (PC) the effect of occlusion was small/medium and significant for both BF (beta = 0.03, SE 0.01, std. beta = 0.37, *p* = 0.01) and PC&BF (beta = 0.03, SE 0.01, std. beta = 0.35, *p* = 0.02). The overall BayesFactor for the effect of occlusion was moderate (BF = 4.41). Bonferroni-Holm corrected pairwise comparisons confirmed significant differences between PC and BF (*p* = 0.047), PC and PC&BF (*p* = 0.01), but not BF and PC&BF (*p* = 0.86). There were no interaction effects (*p*s > 0.79, BF 0.08).

For ball bounce priors, the linear mixed model explained little variance (*R*^2^ = 0.01). The model's intercept was at 0.47 (SE 0.02, 95% CI [0.43, 0.50], *p* < 0.001). Within this model the effect of group was very small and not significant (beta = 0.01, SE 0.03, std. beta = 0.07, *p* = 0.72, BF = 0.13). Relative to the reference category (PC) the effects of BF (beta = − 0.03, SE 0.03, std. beta = − 0.16, *p* = 0.313) and PC&BF (beta = − 0.02, SE 0.03, std. beta = − 0.12, *p* = 0.457) were very small and not significant (overall BF = 0.13). There were also no interaction effects (*p*s > 0.50, BF = 0.06).

### Exploratory analysis

**Fig. 5:**
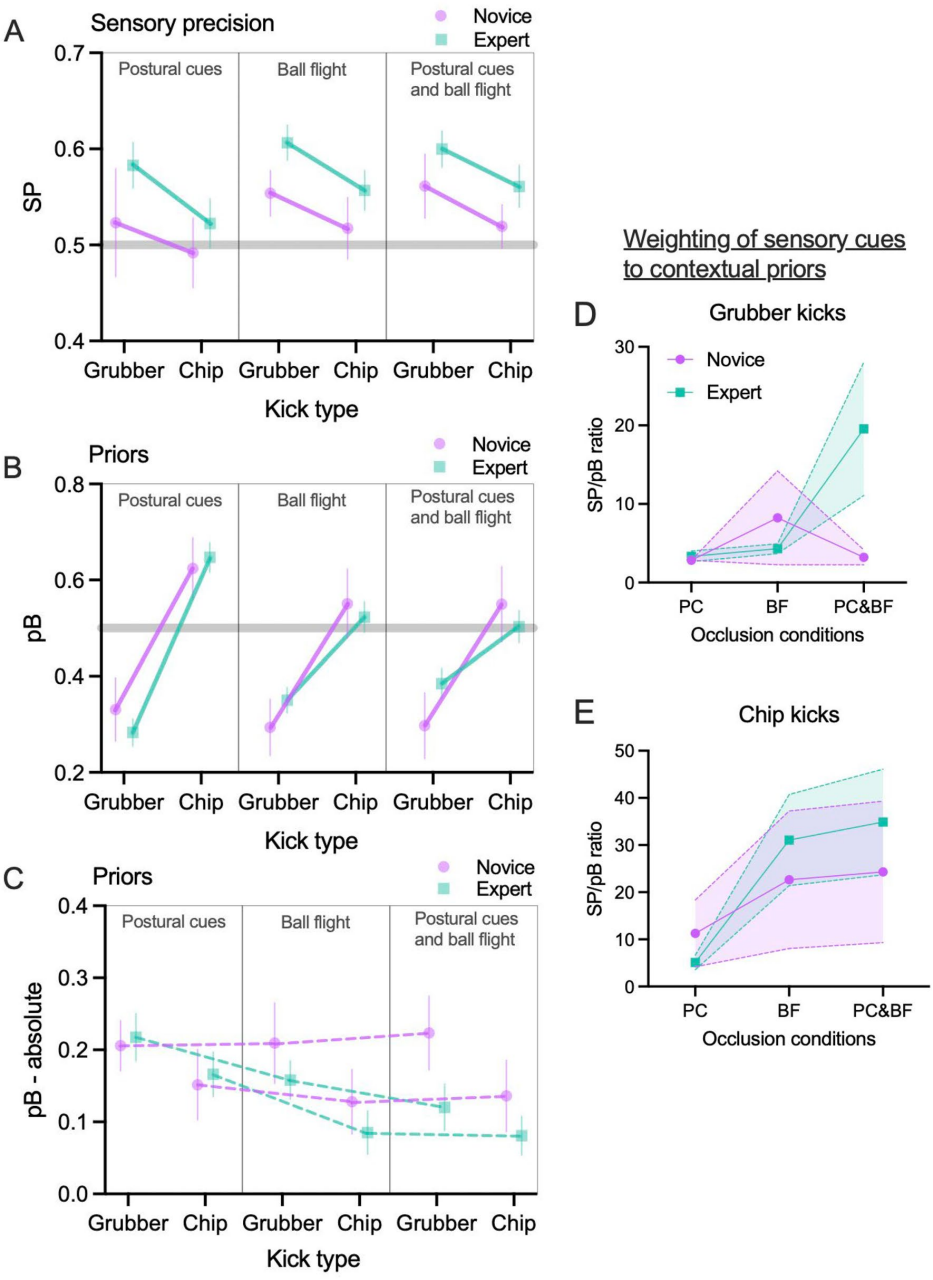
Sensory precision and prior belief parameters across occlusion conditions and kick type (means and 95% CIs). Panels **A** and **B** show means and 95% CIs for the SP and pB parameters from the POMDP model. In panel B, values above 0.5 indicate a belief that lower bounces are more likely and lower values that higher bounces are more likely. Panel **C** illustrates the absolute strength (i.e., deviation from 0.5) of priors across kick type and occlusion condition. Panels **D** and **E** show the ratio of SP to pB values across the conditions (shaded areas represent 95%CIs)

As an exploratory analysis, we added kick type to the statistical model to examine whether participants had different prior expectations of the two kick types, and whether they were differentially sensitive to the pre-bounce cues. For *SP*, kick type was found to be a large and significant predictor when added to the previous model (beta = 0.05, SE 0.02, std. beta = 0.63, *p* = 0.001, BF = 2363.60) (see Fig. 5). The higher SP values for grubber kicks indicated that participants were better able to detect early cues for grubber kicks than chip kicks. The other effects reported above all remained significant in the expanded model.^2^ There were no significant interactions between kick types and the other predictors (*p*s > 0.62, BFs < 1.77).

For pB, the addition of kick type had a considerable effect on the model. The total explanatory power of the expanded model became very large (*R*^2^ = 0.54) with the fixed effects alone explaining 52% of the variance. The model's intercept (corresponding to pB = 0, group = expert, occlusion = PC, and kick type = chip) was at 0.65 (SE 0.02, 95% CI [0.61, 0.69], *p* < 0.001). Within this model, the effect of group was very small and not significant (beta = − 0.02, SE 0.03, std. beta = − 0.13, *p* = 0.50, BF = 0.14). For occlusion conditions, relative to PC, the effect of BF (beta = − 0.12, SE = 0.03, std. beta = − 0.71, *p* < 0.001) and PC&BF (beta = − 0.14, SE 0.03, std. beta = − 0.82, *p* < 0.001) were large and significant (overall BF = 1.34 × 10^4^). Additionally, the effect of kick type was very large and significant (beta = − 0.36, SE 0.03, std. beta = − 2.06, *p* < 0.001, BF = 6.52 × 10^48^), indicating that participants expected higher bounces for grubber kicks than chip kicks.

Large and significant two-way interaction effects were observed for occlusion[BF] × kick type[grubber] (beta = 0.19, SE 0.04, std. beta = 1.09, *p* < 0.001) and occlusion[PC&BF] × kick type[grubber] (beta = 0.25, SE 0.04, std. beta = 1.40, *p* < 0.001). Large and significant three-way interaction effects were observed for group × occlusion[BF] × kick type[grubber] (beta = − 0.16, SE = 0.07, std. beta = − 0.88, *p* = 0.02) and group × occlusion[PC&BF] × kick type[grubber] (beta = − 0.20, SE = 0.07, std. beta = − 1.16, *p* = 0.002). Other interaction effects were not significant (ps > 0.13) (reported in full in supplementary files) (see Fig. 5). Results from Bayesian models supported similar conclusions, indicating strong support for occlusion × kick type (BF = 5.00 × 10^4^) and group × occlusion × kick type (BF = 620.34) interactions, but favored the null for group × kick type (BF = 0.59) and group × occlusion (BF = 0.09) interactions.

To unpick the interaction effects, pairwise comparisons with a Bonferroni–Holm correction were used to compare the differences between priors across occlusion conditions, for each group and each kick type. These tests indicated that the interaction was driven by experts adjusting their priors across conditions, but not novices. For novices, there were no differences between pB across occlusion conditions (*p*s > 0.43). Experts showed a reduction in the weight of the prior from PC to BF (*p* = 0.001) and PC to PC&BF (*p* = 0.001) for chip kicks, and from PC to PC&BF for grubber kicks (see Table 2).

**Table 1:** Description of computational elements for generative model

Model variable	General definition	Model-specific definition
*τ*	Timepoint within a trial	Here there are just two timepoints: a start state, then seeing the bounce cue
*o*_τ_	Observable outcomes at time τ	Start observation High cue Low cue
*s*_τ_	Hidden states at time τ	The agent makes inferences about the hidden state of ball bounce height: High Low
*A *matrix (*p*(*o*_τ_|*s*_τ_))	Matrix encoding beliefs about the relationship between hidden states and observable outcomes (i.e., the likelihood)	The relationship between observed cues and hidden state of ball bounce height
*B* matrix (*p*(s_τ+1_|*s*_τ_))	Matrix encoding how beliefs about states will evolve over time	Encodes the prior belief that either a high or low bounce would occur at *τ* = 2
*D *vector (*p*(*s*_τ=1_))	Matrix encoding beliefs about initial hidden states	Starting prior belief about the prevalence of high and low bounces

**Table 2:** Significance values from pairwise comparisons for prior beliefs across occlusion conditions

Expert	Chip	PC v BF	0.001*
		PC v PC&BF	0.001*
		BF v PC&BF	1.00
	Grubber	PC v BF	0.15
		PC v PC&BF	0.003*
		BF v PC&BF	1.00
Novice	Chip	PC v BF	0.43
		PC v PC&BF	0.44
		BF v PC&BF	1.00
	Grubber	PC v BF	1.00
		PC v PC&BF	0.68
		BF v PC&BF	1.00

Significance values corrected for multiple comparisons (Bonferroni–Holm method). *p < 0.05

## Discussion

Recent work has outlined how applying Bayesian brain perspectives (see Friston et al., 2017; Knill & Pouget, 2004; Rao & Ballard, 1999) to the study of sporting anticipation can help to explain how performers integrate and weight multiple sources of information to make predictions during time-constrained tasks (see Gredin et al., 2018, 2020; Harris et al., 2021). In the present study, we used a Bayesian active inference model of perception to examine observers’ prior beliefs regarding likely ball bounce trajectories and their sensitivity to early postural cues from the kicker and ball flight trajectory. Overall, the responses of both novice and experts in this task were largely driven by responding to online visual cues rather than prior knowledge. Experts were much more sensitive than novices to early postural and kinematic cues from the kicker and flight of the ball but there was little difference in their use of prior knowledge of likely ball bounces. Additionally, experts were observed to rely less on priors when more online information from postural cues and ball flight was available, while novices’ use of priors changed very little when more sensory information became available (see Fig. 5).

Our initial hypothesis, that experts would use both sensory cues and prior knowledge more than novices, was partly supported. Experts showed greater sensitivity to online visual information (std. beta = 1.02, *p* < 0.001) (see Figs. 4 and 5A), but there was no overall difference in use of priors. Figure 4 illustrates that both groups tended towards a belief in higher ball bounces, but these beliefs were similar across experts and novices. Consequently, expertise in this task appears to be characterized by attunement to relevant sensory cues, rather than a strong prior belief in particular bounce trajectories. Previous work in this area (e.g., Gredin et al., 2018) has suggested that experts will integrate both contextual priors and online sensory cues during anticipation. However, there was no explicit contextual information available in this task, only prior knowledge of typical bounce heights. The irregular nature of a rugby ball bounce may mean that even experienced performers did not have a strong prior belief that either high or low bounces were the more likely outcome. Instead, they relied on cues from the kicker and ball flight, from which they were able to make effective inferences about likely bounce outcomes (i.e., *p*(state|observation)). In Bayesian terms, the precision of the distribution of prior beliefs about likely ball trajectories was comparatively low, and therefore was not weighted strongly during anticipation (e.g., Fig. 1). This is an important consideration for the sports anticipation literature that has investigated priors and their importance in the anticipation process (Runswick et al., 2020b). The majority of this work has explicitly provided contextual priors or deliberately developed tasks that are embedded with reliable contextual information (Simonet et al., 2019), potentially up-weighting their importance in the anticipation process compared to the present work.

Based on Bayesian brain accounts of belief updating, we also predicted that when sensory information became more certain, in PC&BF compared to BF or PC occlusion conditions, sensory precision would be higher and priors would be downweighted (i.e., pB values closer to 0.5). The mixed-effects model showed that *SP* values were indeed higher in BF compared to PC (*p* = 0.047), and in BF&PC compared to PC only (*p* = 0.01), but that pB values did not vary across occlusion conditions. Again, this partially supported our initial hypothesis, as SP values were adjusted according to uncertainty, but there was no change in pB. Given the preceding finding—that expertise in the task was primarily related to SP rather than pB—the insensitivity of pB to the occlusion conditions may simply indicate that sensory cues were more important than priors about likely bounce heights for anticipation in this task.

The addition of kick type to the statistical model explained a significant additional amount of variance, as kick type was found to have a large effect on both SP and pB values. Both novices and experts were more attuned to sensory cues (higher SP values) from grubber kicks than chip kicks, regardless of occlusion condition, as is illustrated in Fig. 5A. Novices and experts also expected to observe high bounces for grubber kicks, while pB for chip kicks was close to 0.5, or slightly favored low bounces. Notably, three-way interaction effects between group, occlusion condition, and kick type were also observed for pB values. Follow up analyses indicated that the interactions were driven by experts, but not novices, adjusting the weight of pB in the later occlusion conditions (BF and PC&BF). Experts effectively did not use bounce height priors for chip kicks in BF and PC&BF conditions, and the influence of pB for grubber kicks was similarly reduced from PC to BF to PC&BF. The plot of the absolute pB values (deviation from 0.5; Fig. 5C) further illustrates that the strength of pB was similar across the occlusion conditions for novices, but experts reduced the weight they gave to priors as sensory cues became more certain, as predicted by active inference and Bayesian brain frameworks.

One interpretation of this result is that novices were rigid in their use of information or poor at down-weighting priors when more sensory cues were available. However, Bayesian approaches would predict that a Bayes-optimal weighting of information would lead to a reduced role for priors when the sensory cues were comparatively more certain. Novices, without a developed sensitivity to sensory cues through their generative model, were not very sensitive to kinematic cues even when occlusion occurred later. As a result, both groups were likely displaying ‘Bayes-optimal behaviour’; for experts switching to sensory cues during later occlusion, but for novices continuing to use some prior expectations in an attempt to guide predictions as their sensitivity to cues was low. This result adds to previous findings by Helm et al. (2020) in a handball task where a similar adaptation to the ambiguity of kinematic cues was reported as observers relied more on prior probabilistic information when cues were less reliable. Alongside previous studies adopting a Bayesian approach to anticipation (Gredin et al., 2018, 2021; Helm et al., 2020), the current results support the benefits of this approach in understanding the integration and weighting of multiple information sources to make effective predictions.

The findings from the current study extend knowledge of anticipation in sports by developing an understanding of precision-based weighting of information sources. Previous models of anticipation in sport have pointed to the use of contextual priors and online sensory input (Müller & Abernethy, 2012), discussed issues related to how these information sources are weighted based on reliability (Gray & Cañal-Bruland, 2018; Runswick et al., 2020b), and suggested a Bayesian approach to tackle this question (Gredin et al., 2018; Harris et al., 2021). However, the work presented in this study is the first to combine these narratives in the literature to investigate information integration processes in expert performers using a combination of empirical data and computational modeling. This new approach builds from findings that infer the occurrence of such processes based on self-report from performers (Runswick et al., 2018) to testing explicit predictions using computational methods that can be applied to a variety of important tasks in the domain such as penalty kicks.

## Conclusion

In summary, we aimed to use a Bayesian computational model of perception to examine the relative weighting of priors and sensory cues when anticipating the bounce of a rugby ball. The findings indicated that, in this task, expert observers with extensive task-specific experience were more sensitive to sensory cues but placed little weight on ball bounce priors. When examining priors and sensory precision across different kick types and occlusion conditions it was observed that experts downweighted their priors as later and more precise visual cues emerged. Novices, however, showed little variation in their use of the two information sources, possibly due to their insensitivity to the more precise later visual cues. This work provides an illustration of how Bayesian brain and active inference approaches can be used to understand skilled anticipation in sporting tasks (Arthur & Harris, 2021; Harris et al., 2021) and yields sport psychology with a computational framework by which to understand how performers optimize predictions (Clark, 2013; Friston, 2010; Harris et al., 2021).

## Data Availability

All relevant data and code are available online from: https://osf.io/x64bh/.

## Footnotes

1 For current purposes we use the term ‘contextual prior’ to refer to information sources like game score or opponent’s tendencies. We use the more general term ‘prior’ to include any existing belief that guides behavior, such as knowledge of how balls with different physical properties typically bounce.

2 As the effects in the model remained similar after the addition of kick type they have not been re-reported for brevity, but full details of the expanded model with the addition of kick type are reported in the supplementary files: https://osf.io/x64bh/
